# Effect of Cold Water on the Circulation

**Published:** 1858

**Authors:** H. Bence Jones, W. H. Dickinson


### Effect of the Continued Application of Cold Water Externally Upon the Circulation.

BY DR. H. BENCE JONES AND W. H. DICKINSON, ESQ.

Opportunities of making use of some douche and shower baths of more than ordinary potency having presented themselves, the following experiments were undertaken, with a view of removing some of the uncertainty which now prevails regarding the effects of the outward application of cold water. These experiments are divided into three sections: 1st, on the general effect of the douche or shower bath; 2nd, on the effects of the shower bath at different temperatures; 3d, on the effects of the shower bath in different circumstances.

SEC. 1.—The first experiment was made by a douche bath, by which 225 gallons of water were allowed to fall upon the head for a quarter of an hour. By this the pulse was greatly relaxed in frequency and power, and it became irregular; at one period of the experiment the reduction amounted to 30 beats in the minute. The second experiment was made with a shower bath delivering about 20 gallons of water a minute—upwards of 300 gallons in fifteen minutes. The results were similar to those obtained with the douche bath, but were more marked. During the second minute the pulse was found to be less frequent by 40 beats than it had been previous to the fall of water; and from the fifth minute to the fifteenth, when the experiment terminated, it was observed to be frequently intermitting and very weak. The third experiment was made with a still more powerful shower bath, at Vienna. This delivered nearly 38 gallons of water a minute—upwards of 550 gallons in fifteen minutes, but the openings in the rose were very fine, and the shower was much spread. In the fourth minute the pulse was found to be imperceptible, and during the remainder of the quarter of an hour for which the bath was continued it was feeble and irregular. Afterwards the pulse was observed to be smaller and rather slower than it had been previously, but it was immediately restored by a warm bath. Thus it seems that a strong douche or shower bath produces an excessive immediate effect upon the pulse. By the first shock it may be reduced in rate even 50 beats in the minute; it then recovers a little, but after four or five minutes, when the shivering commences, it again becomes reduced, and often is rendered quite imperceptible.

Sec. 2.—The experiments in this section were made for the purpose of showing whether the effect varied with the temperature of the water. The most interesting are two which were made with the powerful shower bath alluded to in section 1, second experiment. In the first, the water was at 70 deg. Fahrenheit. The pulse did not fall in rate for three minutes, although it lost much in strength and volume. When shivering commenced, at the end of the fourth minute, the pulse was imperceptible, and it was scarcely to be felt until the end of the sixth, and it remained weak and irregular until the termination of the experiment at the end of the tenth minute. In the second experiment the water was iced down to 50 deg. F. The effect was much more rapid. During the first fifteen seconds the pulse was reduced at the rate of 38 beats per minute; this was followed by a reaction better marked then before, and the annihilation of the pulse, which followed the commencement of shivering, was much more complete and of longer duration.

Sec. 3.—Some of the effects observed to follow the use of the shower-bath, taken under varying circumstances, are here stated. Two experiments were made: one at the baths at Ischel, in Austria, and one at the Prussian bath, at Vienna, where cold shower-baths were alternated with very hot vapor-baths. It was found that the increased action of the pulse produced by exposure of the body to hot steam prevented that depression which would otherwise have resulted from the cold water. A converse experiment is quoted from Dr. Currie's "Medical Reports." An ague patient, who had derived advantage from the cold effusion during the hot stage of the fit, nearly died from the alarming depression which resulted from the same application while he was in the cold stage.

The general conclusions are—

1. The useful effect of a strong douche or shower bath is the immediate depression of the pulse. By the first shock of water between 64 deg. and 68 deg. F. the pulse becomes weak and irregular, and may be reduced in rate even fifty beats in the minute. After the first shock the pulse recovers a little, but remains weak until the secondary effect or showering comes on, when it becomes weaker and intermitting, and may be quite imperceptible. After ten to fifteen minutes the pulse remains very small and weak, and shivering continues while the experiment lasts.

2. If the shower bath is a small one, (eight gallons,) and the person taking it in good health, no great difference is perceived in the pulse whether the water is hot (110 deg.) or warm (74 deg. F.) If the water is very cold, (47 deg. F.) the pulse becomes smaller, but the rate is not affected.

With a shower-bath giving twenty gallons per minute a difference of twenty degrees (from 70 deg. to 50 deg. F.) causes a great difference in the shock. The difference in the after-effect, or shivering, is not so marked. The depression of the pulse when the shivering comes on is more continuous with the colder water, and is manifest up to the end of the experiment.

3. When the pulse is raised above, or depressed below, its healthy standard, the shower-bath or douche produces very much less or a much greater effect than would be produced by the bath under ordinary circumstances.

As it seemed possible that a part of the reduction of the pulse might be due to the action of the cold water upon the capillaries and the radial artery in which the pulse was felt, a set of experiments were made in which the forearm and hand were exposed to temperatures varying from 25 deg. to 124 deg. F. The results of these experiments may be thus stated:—

1st. When one arm is in water at 50 deg. and the other in air at 46 deg., no difference in the pulse is observed in fifteen minutes.

2d. When one arm is in the water at 110 deg. and the other in air at 46 deg. F., little if any difference could be felt in the same time.

3d. When one arm is in water at 44 deg. and the other in water at 107 deg. F. there was the same result in the same time.

4th. Even one arm at 33 deg. and the other at 112 deg. give no result.

5th. Still lower and higher temperatures, 25 deg. and 115 deg. F., did not give any decided results in fifteen minutes.

6th. The douche-bath on the arm and hand, at 42 deg. produced no greater effect on the pulse than still water at 44 deg. F.

Hence, generally, it follows, that no part of the effect produced by the shower-bath on the pulse, depends on the action of the water on the hand and forearm in which the pulse is felt. —*Proceedings of Royal Med. and Chirurgical Society*, April 14th, 1857.

